# An Algorithm for Testing the Efficient Market Hypothesis

**DOI:** 10.1371/journal.pone.0078177

**Published:** 2013-10-29

**Authors:** Ioana-Andreea Boboc, Mihai-Cristian Dinică

**Affiliations:** 1 Financial Engineering Section, Swiss Finance Institute at École Polytechnique Fédérale de Lausanne, Lausanne, Switzerland; 2 Department of Finance, Bucharest University of Economic Studies, Bucharest, Romania; Cinvestav-Merida, Mexico

## Abstract

The objective of this research is to examine the efficiency of EUR/USD market through the application of a trading system. The system uses a genetic algorithm based on technical analysis indicators such as Exponential Moving Average (EMA), Moving Average Convergence Divergence (MACD), Relative Strength Index (RSI) and Filter that gives buying and selling recommendations to investors. The algorithm optimizes the strategies by dynamically searching for parameters that improve profitability in the training period. The best sets of rules are then applied on the testing period. The results show inconsistency in finding a set of trading rules that performs well in both periods. Strategies that achieve very good returns in the training period show difficulty in returning positive results in the testing period, this being consistent with the efficient market hypothesis (EMH).

## Introduction

This paper describes a genetic algorithm used to create a trading system, consisting of several rules for opening and closing trading positions in the FX market. The aim of this study is to assess the weak form efficiency of the EUR/USD market. Our paper shows that the distribution of the outcome in the out-of-sample period is uniformly distributed around an average close to 0. This provides evidence that all the information available in the EUR/USD market is reflected in the price and no arbitrage can be made by trading this currency pair based on historical information.

Our findings should capture the attention of investors in the FX market that base their decisions on technical analysis signals. The results are in the support of previous academic literature that in general provides evidence for the impossibility of forecasting financial market movements by only analyzing historical prices.

Algorithmic trading has evolved exponentially in recent years because of more rapid reactions to temporary mispricing and easier price management from several markets [1]. As compared to human dealers, computers can learn from thousands of sources of information simultaneously and avoid emotional influence.

Technical analysis is a methodology of forecasting price movements by analyzing past market data [2]. The efficient market hypothesis (EMH) [3] contradicts this approach by stating that all public information in the market is immediately reflected in prices; therefore, no arbitrage can be made based on historical data. Time series is split in two parts. The trading system with several parameters is applied in-sample over the training period and strategies that generate the highest returns are selected and tested over the following period (out-of-sample). The objective of the system is to achieve high returns over the testing period. The impossibility of finding a good performing strategy over both training and testing period would support the EMH.

The research proceeds as follows. This section offers a review of the existing literature regarding the tests on the efficient market hypothesis, studies on the performance of technical analysis based on several indicators as well as the improvement of trading strategies using genetic algorithms. Section 2 presents the database used for testing the efficiency of the system and the methodology involved. Section 3 discusses empirical findings of our analysis and concludes. One currency pair has been used, EUR/USD.

### Efficient Market Hypothesis

EMH, developed by Eugene Fama [3], assumes that all the information in the market at a specific moment is reflected in the prices and therefore market participants cannot consistently perform better than the average market returns on a risk-adjusted basis. However, empirical findings have shown that the EMH may be questionable. Hasan et al. [4] find inefficiencies in the Dhaka stock market. They notice that factors like return, market capitalization, book-to-market ratio and market value influence the share returns. Moreover, similar features such as thin trading, volatility, small number of securities listed and investors’ attitude towards investment strategy characterize DSE, as well as other emerging markets.

Several studies find volatility in the level of efficiency over time and among different markets. Alvarez-Ramirez et al. [5] observe that the efficiency degree of financial markets changes over time. The relative efficiency of the US stock market varied over 1929–2012, with a decline in the late 2000s induced by the economic recession. The most efficient period was 1973–2003. Another study showing that the degree of inefficiency is not constant over time is made in [6]. IRR/USD market was inefficient over 2005–2010 and this may be caused by the negative long-range dependence, meaning that if the exchange rate is up it is likely to go down in the close future. A similar result is revealed by Kim et al. [7]. They provide evidence that supports time-varying return predictability of the Dow Jones Industrial Average index over the period 1900–2009. While the market seems efficient during market crashes, economic and political crises induce predictability in returns. The efficiency of the Asian stock markets varies with the level of equity market development [8]. The developed emerging markets are found to be weak-form efficient, while the secondary emerging markets are characterized by inefficiencies.

Dragota et al. [9] could not reject the weak-form EMH for the Bucharest Stock Exchange by applying Multiple Variance Ratio test to random walk hypothesis. For the same market, Armeanu and Balu [10] tested the efficiency of the Markowitz model, emphasizing the benefits of portfolio diversification. Charles et al. [11] evaluated the predictability of exchange rate returns and found that while they are unpredictable most of the times, return predictability may appear with coordinated central bank interventions and financial crises. The Chinese stock markets efficiency is investigated in [12]. The results show that Class A shares, which are generally available for domestic investors, seem efficient, while Class B shares, eligible for foreigners, are significantly inefficient. Trolle and Schwartz [13], using a database of 11 years of data for crude oil and natural gas futures and options traded on NYMEX, found that it is difficult to explain the variation and the level in energy variance risk premia using systematic factors such as the returns on commodities or equity market portfolios or specific factors such as inventories.

### Technical Analysis

Most automated trading systems use several indicators in order to generate purchase and sale recommendations [14]. One found that the best indicator for companies with high capitalization is RSI and the best for small capitalization companies is Momentum. Moreover, indicators that do not give many trade signals, such as Momentum, are more suitable when the transaction costs are high. One research assessed the performance of technical analysis in the US equity market for some technical industry sectors and small caps, over the period 1995–2010 [15]. Results show that the strategies are capable of outperforming the buy-and-hold strategy after adjusting for data-snooping bias and without transaction costs in the first half of the sample period. However, the same strategies are not able to produce superior performance over the second half. Success in the period 1995–2002 is tempered when introducing transaction costs. Moreover, the forecast of short-term return became weaker in the recent years, this being consistent with the EMH in the equity market. A positive performance of technical analysis is generated by applying moving average trading rules on 16 European stock markets over the period 1990–2006 [16]. A moving average trading rule combined with a strategy that at buy signals recommends investing in the stock market, while at sell signals recommends investing in the money market outperforms the buy-and-hold strategy over the sample period.

In [17] is found that one can achieve performing returns using trading strategies only if he has full information of the stock price change for the future. However, if the future information is not accurate, it can be useless in increasing profits. Moreover, a search in a strategy space to get high profit is impossible and this is based on lack of future information of a company.

Trading strategies have been mainly based on technical analysis in the commodity futures market [18], [19], [20] and foreign exchange market [21], [22], [23], [24]. Evaluation of the technical analysis’ performance in the equity markets has generally been done using market indices such as Dow Jones Industrial Average [25], [26], S&P 500 [26], [27], NYSE and NASDAQ [26], [28], [29] or Russell 2000 [26], [27], [29]. Technical analysis has evolved beyond filter and moving averages rules, now including psychological barriers such us resistance and support levels [30]; [31].

### Genetic Algorithms

In recent years, individuals and companies have developed algorithms that try to improve profitability of trading rules. Genetic algorithms (GA) represent a class of optimization techniques that generate solutions to search problems and quickly adapt to changing environments. GA were developed by Holland [32] and they simulate the process of natural evolution. As the species evolve through genetic processes such as selection, crossover and mutation, GA create classes of solutions that evolve over some generations through analogous processes in order to generate one solution with the best fit to the specific problem [33]. Algorithms start by creating some strategies with specific parameters. In the following steps, they dynamically change their parameters in order to achieve higher profits.

In a natural evolution process, species change over time. New organisms are born by recombination between members. They inherit parents’ traits and are also influenced by environment conditions. The natural selection process comes from the fact that while the population grows, the organism need to struggle for resources. Therefore, only the organisms that possess well-suited characteristics for this struggle will bring more offspring to the new generation.

Holland [32] developed a way in which the natural evolution process might be imported into algorithms that offer solutions to search problems. GA are very suitable for managing financial markets because these represent a continuous changing environment and trading strategies need to adapt to the new conditions. The search problem is represented by finding a strategy that achieves positive excess returns when applied to a specific sample. GA generate many strategies and those well fitted (according to a specific function that can be mean return, Sharpe ratio or one that takes into account also environment conditions) are selected for passing in the new generation and for recombining to generate new strategies.

Mendes et al. [34] developed a system based on a genetic algorithm that optimizes a set of rules to obtain a profitable strategy to trade EUR/USD and GBP/USD. The system generates individuals defined by ten mandatory and optional rules, from which five of them decide whether opening a long/short position or not at current price in the market and the other five decide when to close an opened position. The rules contain 31 parameters that evolve in many generations through selection, crossover and mutation and, based on return and risk, the individual that had the highest performance is selected and tested in the next period. Results have shown that, considering transaction costs, the best individuals in the training series were often not able to achieve positive results in the out-of-sample test series. Dempster and Jones [2] created an adaptive trading system that uses genetic programming. They used USD/GBP spot foreign exchange tick data from 1994 to 1997. The algorithm is applied on out-of-sample data to provide new rules and a feedback system helps rebalancing the rule portfolio. The genetic algorithm is profitable even in the presence of transaction costs.

Another study about the performance of the genetic algorithms for FX markets has been developed in [35]. The authors show that the system often returned profit when the testing period was consecutive to the training period. They concluded that the success of the system depended on the similarity in the trends of the two periods. Also, genetic algorithms succeeded in finding performing trading rules for six exchange rates over the period 1981–1995 [36].

One bias that may appear when one tests a large number of strategies on the same sample is the data-snooping bias. As explained in [37], data-snooping bias appears when a set of data is used more than once for the purpose of model selection. Strategies that generate positive returns on a specific sample may be performing only due to luck and do not have a genuine predictive power. Therefore, when applied to a different sample, the results can be negative and investors may suffer important losses. A solution to this problem is the Bootstrap Reality Check developed by White [38] that relies on resampling the return series in order to give a reliable verdict regarding the genuine performance of the strategy.

## Materials and Methods

The database used in this paper is the tick-by-tick series of EUR/USD currency pair over the year 2012 (ratedata.gaincapital.com). Time series with frequency of 60 minutes were used for testing the performance of the genetic algorithm.

Time series have been separated in two sets: the training period and the testing period. The first one considers the first six months of 2012 and is used for finding the strategy that achieves the highest performance. The second set tests the performance of the strategy found in the first step.

The algorithm is applied 100 times on the training time series, in order to find the characteristics of the best 100 individuals. We then assess the performance of these individuals on the out-of-sample series.

The hourly data extracted from the tick-by-tick data also consider the minimum and maximum tick for both bid and ask quotes. We needed this information to establish if the take-profit or stop-loss level had been reached during that period.

The purpose of the genetic algorithm is to optimize a set of trading rules to generate higher profit. Trading rules base their decisions on several indicators presented below together with their formulas.

1 Exponential moving average. It gives greater weights to the latest prices when computing the average. When the price is above this indicator, the signal is for a long (buying) position and when the price is below the exponential moving average it signals the selling.
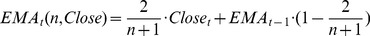
(1)Where *EMA* is the exponential moving average indicator, *n* is the number of periods and *Close* is the closing price of the period.
2 Moving Average Convergence Divergence – it is an indicator based on several other technical analysis indicators. Firstly, the *MACDline* is computed as a difference between two exponential moving averages. Secondly, we compute a signal line as an exponential moving average of the *MACDline.* Finally, the MACD indicator is computed as the difference between the *MACDline* and the signal.
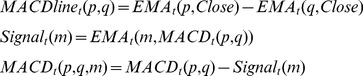
(2)Where *p* is the number of periods of the short exponential moving average, *q* is the number of periods of the long exponential moving average, *Close* is the closing price of the period and *m* is the number of periods of the moving average of the *MACDline*.

This indicator offers buying or selling signals when its value is positive, respectively negative.

3 Relative Strength Index – is a technical analysis indicator that gives overbought and oversold signals. The overbought signal is given when the RSI value is over a specific benchmark (usually 70 or 80) and the oversold signal is given when this value is under another benchmark (the standard is 20 or 30).
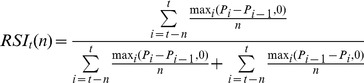
(3)Where P is the closing price of the period and n is the number of the periods used to compute the RSI.

Further, we start the description of the algorithm with the definition of an individual.

### The Individual Characteristics

In a genetic algorithm for setting a FX trading system, each individual is represented by a set of technical analysis rules. Each rule can be considered as a chromosome, while the parameters that define a rule are considered genes. Here we consider the individual as being defined by 6 chromosomes (rules) and 24 genes (parameters). The rules are divided in 4 rules that set the conditions for opening a position and the rest 2 rules are those that define the conditions for exiting the position. Each rule contains a Boolean gene that can activate or deactivate the rest of the rule’s genes.

Following, are described the rules (chromosomes).

### Rules for Position Opening

**Rule 1. Exponential Moving Average: EMA(n).** Genes:

1 Boolean_EMA –takes the values 0 or 1. Value 0 deactivates the rule, while value 1 activates it.
2 Nr_periods_EMA, noted *n*, takes values between 5 and 90.

The rule generates trades as follows. If there is no current open position, then a long position is generated only if the close price is higher than *EMA(n)* and a short position is generated only if the close price is lower than *EMA(n)*. If a position is currently open, then this rule is ignored.

**Rule 2. Moving Average Convergence Divergence: MACD(p,q,m).** Genes:

3 Boolean_MACD – takes the values 0 or 1. Value 0 deactivates the rule, while value 1 activates it.
4 Periods_short_MA, noted *p* takes values between 5 and 90
5 Periods_long_MA, noted *q* - takes values between 10 and 100, with the restriction *q* >*p*
6 Periods_signal_MACD, noted *m* - takes values between 5 and 25 and is the moving average of the difference between the short and the long moving average.
7 Boolean_signal - takes the values 0 or 1. Value 0 sets the value for the signal to 0. Basically, it transforms the MACD in a simple rule of moving averages crossover. Value 1 activates the signal. For the 0 value is attached a probability of occurrence of 25%, while for value 1 the probability is set to 75%

The trades are generated by this rule as follows. Firstly, if a position is already open, the rule is ignored. If there is no currently open position, then, if the Boolean_signal has the null value, the rule takes into consideration only the short and the long moving averages. Therefore, a long position is opened when the short moving average is higher than the long one and a short position is opened otherwise. If the Boolean_signal takes the value 1, the rule proceeds as follows. If the difference between the short moving average and the long one is higher than the value of the signal, then a long position is opened, while otherwise is opened a short position.

**Rule 3. Relative Strength Index: RSI (n).** Genes:

8 Boolean_RSI – takes the values 0 or 1. Value 0 deactivates the rule, while value 1 activates it.
9 Periods_RSI, noted *n*, takes values between 5 and 50.
10 Oversold_signal_RSI, noted *p*, takes values between 15 and 35.
11 Overbought_signal_RSI, noted *q*, takes values between 65 and 85.
12 Boolean_signal_RSI - takes the values 0 or 1. The use of this gene is described below.

The rule generates trades only if currently there is no open position. The trades are generated based on the Boolean_signal_RSI value as follows. When it takes the value 0, a long position is opened when the RSI value drops under *p* and a short position is opened when the RSI value rises over *q*. When it takes the value 1, a long position is opened when the RSI value rises over *p* and a short position is opened when the RSI value drops under *q*.

**Rule 4. Filter(n).** Genes:

13 Boolean_Filter –takes the values 0 or 1. Value 0 deactivates the rule, while value 1 activates it.
14 Periods_Filter, noted *n*, takes values between 1 and 15.
15 Increase_signal_Filter, noted *p*, takes values between 50 and 100 pips.
16 Decrease_signal_Filter, noted *q*, takes values between 50 and 100 pips.
17 Boolean_trader_type_Filter - takes the values 0 or 1.

This rule respects the same restriction as the rest of the three opening rules: if there is already a currently open position, the rule is ignored. The trades are generated based on the Boolean_ trader_type_Filter value as follows. Value 0 signals a trend follower (enters long if the price increases more than *p* pips or short if the price decreases more than *q* pips). Value 1 signals that the trader will enter long if the price decreases more than *q* pips or short if the price increases more than *p* pips.

For the above, a great importance have the Boolean genes that activate or deactivate the rules: 1, 3, 8 and 13. When all of them take the value 0, this means that the individual will never open a position (because no opening rule is active). In order to avoid such situations, that have a probability of occurrence of 6.25%, we proceed the following way. If these genes take all value 0, then we randomly change the value for one of them.

Moreover, if two or more of these genes take simultaneously the value 1, then a position is opened only if all the active rules give the same trading signal (to buy or to sell). Therefore, it is more probable that an individual that has only one active rule to trade more than an individual that has all the rules active.

As important as the rules that define the conditions to open a position are the rules used to exit that position, in order to take the profit or to cut the losses. Following are described these rules.

### Rules for Exiting the Position

**Rule 5. Fixed exit levels (p,s).** Genes:

18 Boolean_fixed_exit –takes the values 0 or 1. Value 0 deactivates the rule, while value 1 activates it.
19 TP_fixed, noted *p*, takes values between 15 and 150 pips
20 SL_fixed, noted *s*, takes values between 10 and 100 pips

Opposite to the opening rules, the rules for exiting the position are active only when a position is opened. The above rule exits a long position if the price rises at least *p* pips (take profit) or drops at least *s* pips (stop loss). Accordingly, the rule exits a short position if the price drops at least *p* pips (take profit) or rises *s* pips (stop loss).

**Rule 6. Trailing exit levels (p,s,q).** Genes:

21 Boolean_trailing_exit - takes the values 0 or 1. Value 0 deactivates the rule, while value 1 activates it. This gene is conditioned by gene number 18. If gene 18 takes value 0, then gene 21 takes value 1 and if gene 18 takes value 1, then gene 21 takes value 0.
22 TP_trailing, noted *p*, takes values between 15 and 150 pips
23 SL_trailing, noted *s*, takes values between 10 and 100 pips
24 Trailing_level, noted *q*, takes values between 10 and 100 pips, under the restriction that *q*<*p*.

The above rule can be active only if a position is already open and rule 5 is not active. If a long position is already open and the price rises at least *q* pips, but less than *p* pips, the take profit and stop loss levels are updated, by increasing them with *q* pips. Continuing, if the price rises another *q* pips, but the new take profit level is not reached, then the stop loss and take profit levels are updated again, by increasing them with another *q* pips. The same procedure is followed until the stop loss is reached or during one period the take profit is hit. In the case of a short position, same methodology is used, with the difference that the stop loss and take profit levels are updated by decreasing them with *q* pips.

### The Genetic Algorithm

After defining the individual, characterized by the rules for entering into position (based on the technical analysis indicators) and by the exit rules (take profit and stop loss), we proceed to the genetic algorithm, which consists in the following steps:

1. A population of 100 individuals is randomly generated.
2. We compute the profit or loss generated by each individual over the training period. Each individual is evaluated based on this measure.
3. The individuals are ranked based on the generated profit or loss.
4. The new generation is created by the following procedures:
  - *Elitism.* The most profitable individual is automatically passed to the new generation
  - *Selection of parents.* The probability of a given individual to become a parent for the new generation is based on its ranking. In order to increase the computational speed, we divided the individuals in 10 classes of fitness (profitability). First class contains the first 10 best-ranked individuals, the second class contains the individuals ranked 11th to 20th, while the 10th class contains the last 10 ranked individuals (Table 1). For the individuals of the same class, we attach the same probability. In addition, the probability is higher for classes that contain better-ranked individuals (e.g. the first class will have attached a higher probability than the 10th class).
  - *Crossover.* Using the selection criteria described above, pairs of two parents are randomly chosen. Each pair of parents will create a new individual. In order to choose what genes from what parent will be passed to the new individual, a number *n* (where 1<*n*<24) is randomly generated. The new individual will receive the genes 1 to *n* from one parent and the genes *n*+1 to 24 from the other parent. The gene 21 will still depend on the gene 18. This way 80 individuals from the new generation are obtained.
  - *Introduction of migrants.* In order to increase the diversity and to avoid fast convergence, we randomly generate 19 individuals in the new generation.
5. The new generation becomes the actual generation and the steps 2–4 are repeated.
6. We repeat the procedure from steps 2–5 until we reach 30 such iterations.

**Table 1:** Probabilities of individuals to become parents.

Class	Number of individualsin the class	Probability for eachindividual in the class	Classprobability	Cumulativeprobability	Cumulativepopulation
1	10	3.00%	30.00%	30.00%	10
2	10	2.00%	20.00%	50.00%	20
3	10	1.25%	12.50%	62.50%	30
4	10	1.00%	10.00%	72.50%	40
5	10	0.75%	7.50%	80.00%	50
6	10	0.50%	5.00%	85.00%	60
7	10	0.45%	4.50%	89.50%	70
8	10	0.40%	4.00%	93.50%	80
9	10	0.35%	3.50%	97.00%	90
10	10	0.30%	3.00%	100.00%	100

Notes: Table 1 shows the probabilities of individuals to become parents in the crossover step of the genetic algorithm. The probabilities are sorted by the cumulative profitability in the training period and, in order to increase the computational speed, the individuals are divided in 10 classes of fitness.

By executing the genetic algorithm, is obtained one individual, the result of the evolution. We repeat the genetic algorithm for 100 times in order to obtain 100 such individuals (sets of trading rules). Then, these 100 best individuals from the training period will be evaluated on the testing period. The evaluation procedure consists in assessing the profit or loss (expressed in pips) generated by the each individual in the testing period. The results obtained are attached in the Supporting Information file.

GA was developed under Eclipse Integrated Development Environment (IDE) version Helios Service Release 1 using Java Development Kit (JDK) version “1.7.0_21″. Three Java Archive (JAR) libraries have been added to the project: JFreeChart (http://sourceforge.net/projects/jfreechart/files/1.%20JFreeChart/1.0.14/) and JCommon (http://sourceforge.net/projects/jfreechart/files/3.%20JCommon/1.0.17/), both used to plot the cumulative profits of strategies and CSV_JAR ((http://www.java2s.com/Code/JarDownload/opencsv/opencsv-2.3.jar.zip)) used to read the data from comma-separated values (CSV) files.

## Results and Discussion

**Figure 1:**
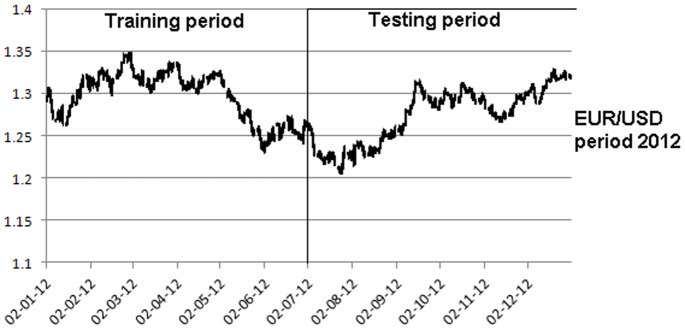
EUR/USD over the period 2012. Figure 1 represents the evolution of the EUR/USD pair over the year 2012. The first half shows the training period, while the second shows the testing period.

To analyze the results, we firstly discuss the evolution of EUR/USD in the training and testing period (Fig. 1). During the training period, a short upward movement, followed by a sideways evolution, firstly characterizes the exchange rate. Starting with May 2012, a strong downward trend is set. The testing period starts with a continuation of the downward trend, followed by a reversal and an upward trend in August 2012. The final part of the testing period is characterized by a sideways evolution of the EUR/USD exchange rate. Both training and testing period contain price movements in trend or sideways. Therefore, it is expected that the rules that perform relatively well in both types of markets (trending and sideways) will obtain good results in both periods.

**Figure 2:**
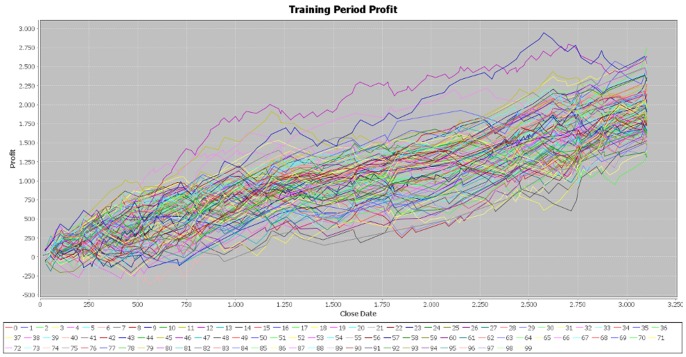
Profit on the training period – simulation 1. Figure 2 is the outcome obtained in the first simulation by applying the genetic algorithm on the EUR/USD pair over the training period (first half of the year 2012).

**Figure 3:**
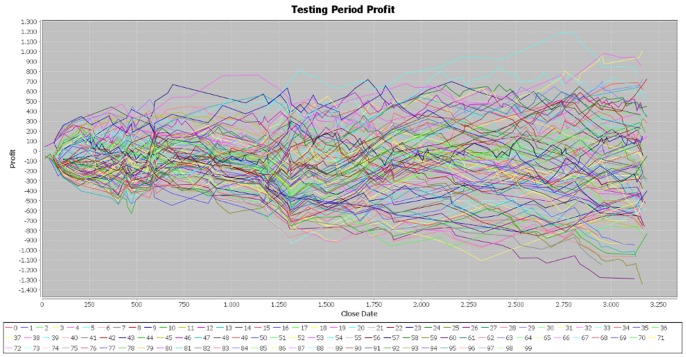
Profit on the testing period – simulation 1. Figure 3 is the outcome obtained in the first simulation by applying the genetic algorithm on the EUR/USD pair over the testing period (second half of the year 2012).

The cumulative profit exhibits an upward trend on the training period for all the 100 best individuals (Fig. 2). The increase in the cumulative profit does not have important variations, showing that the individuals are well fitted on the training period. However, on the testing period, the cumulative profit seems uniformly distributed around the null value and its dispersion increases with time (Fig. 3). The individuals that performed best on the training sample are not able to achieve similar results on the testing sample, providing evidence that EUR/USD market is weak-form efficient.

**Figure 4:**
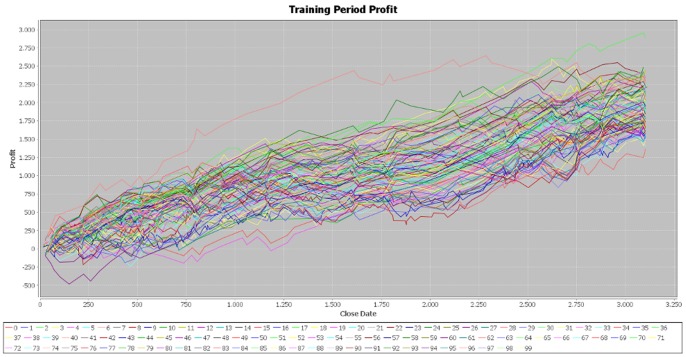
Profit on the training period – simulation 2. Figure 4 is the outcome obtained in the second simulation by applying the genetic algorithm on the EUR/USD pair over the training period (first half of the year 2012).

**Figure 5:**
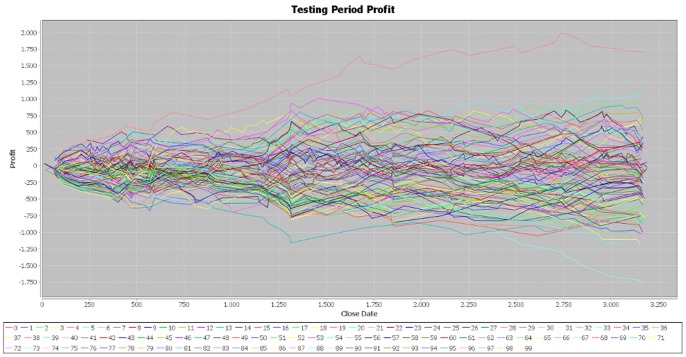
Profit on the testing period – simulation 2. Figure 5 is the outcome obtained in the second simulation by applying the genetic algorithm on the EUR/USD pair over the testing period (second half of the year 2012).

**Figure 6:**
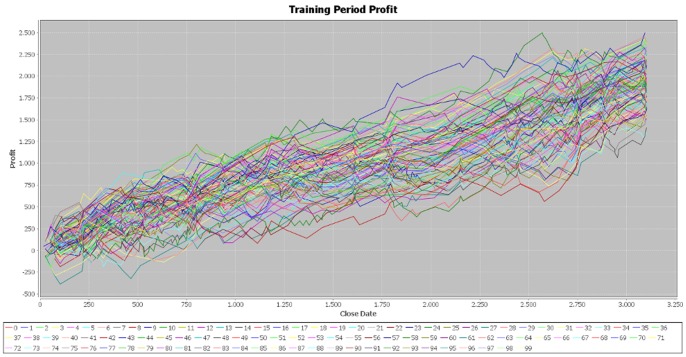
Profit on the training period – simulation 3. Figure 6 is the outcome obtained in the third simulation by applying the genetic algorithm on the EUR/USD pair over the training period (first half of the year 2012).

**Figure 7:**
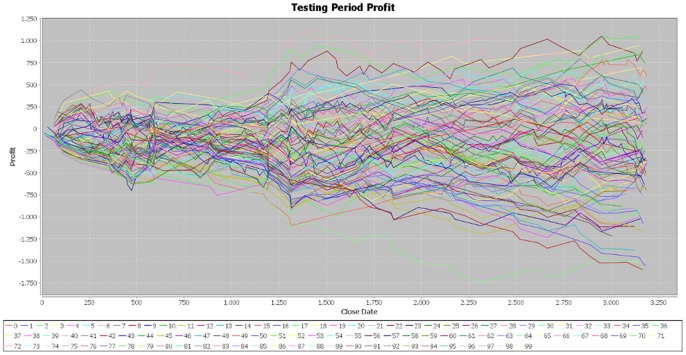
Profit on the testing period – simulation 3. Figure 7 is the outcome obtained in the third simulation by applying the genetic algorithm on the EUR/USD pair over the testing period (second half of the year 2012).

We made two more simulations of the program in order to verify the consistency of our results and the parameters of the generated individuals are attached in the Materials S1 file, together with those of the first simulation. In the case of the second simulation, the results for the training period are very similar to those obtained in the initial one (Fig. 4). In addition, the cumulative profit over the testing period exhibits the same pattern of the first simulation (Fig. 5). By running the third simulation, the results are very similar (Fig. 6, Fig. 7). Therefore, these simulations validate the initial results that the best performers over the training period are not able to achieve similar results over the testing period. Our results are consistent with those obtained by Mendes et al. [34], suggesting the weak-form efficiency of the EUR/USD market.

Next, we computed the statistics of all the 300 generated individuals for the training and testing periods. Statistics with and without transaction costs are computed. Results are similar in both cases.

Statistics on the training sample show that the minimum, maximum and average cumulative profit are all positive and high (Table 2). This happens because each selected individual is the most profitable from a set of 3000 individuals. Therefore, their outcome is predictable high.

**Table 2:** Statistics for the cumulative profit on the training period.

Type of profit	Average	Median	Min	Max	StDev	Nr Profitable	Skewness	Kurtosis	JB	p-val
Real	1,900.05	1,876.10	1,314.00	2,898.00	284.15	300	0.49	2.95	12.25	0.0022
Without cost	2,007.83	1,968.65	1,436.40	2,967.70	275.93	300	0.57	3.13	16.33	0.0003

Notes: Table 2 shows the statistics for the cumulative profit expressed in pips on the training period. The statistics are computed for both cases: with and without transaction costs.

**Figure 8:**
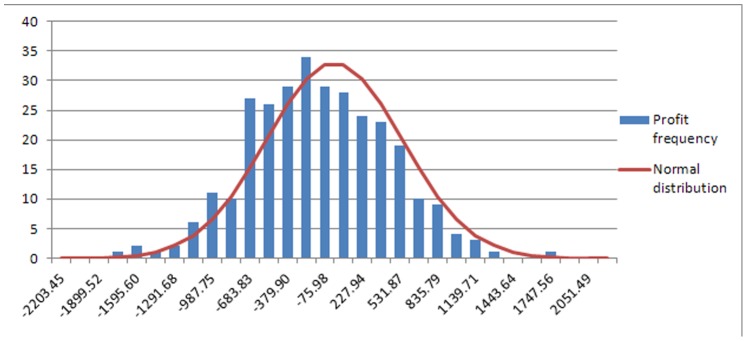
Distribution of the profits on the testing period. Figure 8 shows the distribution of the outcome obtained in all the three simulations on the testing period. The normal distribution with mean 0 and standard deviation equal to the one of the empirical distribution of the profits is also represented.

The second period is a robustness test for the strategies found in the first period. The average cumulative profit at the end of the testing period is negative, but close to 0, being consistent with efficiency hypothesis that no arbitrages can be made using the winning strategies from period 1 (Table 3). In addition, the variability of the outcomes is higher in the testing period (the standard deviation is almost double in the testing period than in the training one). The values of the Skewness and Kurtosis statistics provide evidence that the profit distribution over the testing period may be normal. The empirical distribution plotted in Fig. 8 shows that the profits follow a distribution close to the normal one, but it is skewed from the standard normal distribution due to its negative average.

**Table 3:** Statistics for the cumulative profit on the testing period.

Type of profit	Average	Median	Min	Max	StDev	Nr Profitable	Skewness	Kurtosis	JB	p-val
Real	−198.94	−224.00	−1,780.00	1,702.00	550.86	107	0.07	3.04	0.26	0.8788
Without cost	−109.94	−130.85	−1,672.90	1,749.60	560.75	126	0.05	2.92	0.22	0.8978

Notes: Table 3 shows the statistics for the cumulative profit expressed in pips on the testing period. The statistics are computed for both cases: with and without transaction costs.

Further, we have applied Jarque-Bera test in Eviews 7 to check for normality. The JB statistic is computed as:
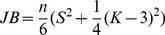
(4)Where *n* is the number of observations, *S* is the skewness and *K* is the kurtosis. The test is built on a joint null hypothesis of a skewness of 0 and a kurtosis of 3 because these values characterize the normal distribution. As shown in Table 3, the null hypothesis of normal distribution for the testing period profits cannot be rejected.

A frequent problem met in the case of technical trading rules is the data-snooping bias. It may appear when more strategies are tested on the same sample. In this way, a rule may be performing in a period only due to luck. Therefore, when it is applied to another period, it generates negative returns. In the literature, a data-snooping test is applied to check for the validity of good performance. In our case, the out-of-sample results are distributed around 0, showing that in the case of the EUR/USD market one cannot find an outperforming strategy based on historical prices. Therefore, in the absence of a consistently profitable strategy (genuine or due to luck), the data-snooping test is not needed in our algorithm.

Concluding, our results show that the hypothesis of weak-form efficiency cannot be rejected in the case of EUR/USD market. Of course, this does not necessarily mean that one cannot prove the market inefficiency by finding a set of rules that consistently achieve profits. However, finding this set of rules represents a difficult task. We consider that our main results suggest that an investor should carefully analyze before taking speculative positions based on technical indicators and computer-based algorithms because there are higher chances to loose on the long-run. The fact that a sophisticated algorithm was not able to achieve sustainable profits supports our remark.

We recommend as future research adding some filters to the trading strategies in order to avoid false signals. For example, a strategy may achieve better results if the investor enters a position only after receiving the same signals for several periods. The same filter can be applied for the exit rules. Moreover, if some strategies are found to be performing, a data-snooping test should be applied in order to check their genuine predictive power.

## Supporting Information

Materials S1 **This file contains the parameters of the individuals generated by the genetic algorithm.** There are three sheets, each one containing the parameters (genes) of the individuals generated in each simulation. The first sheet contains the genes’ values of the 100 individuals generated by the first simulation. The second and the third sheet contain the genes’ values of the individuals generated by the additional two simulations.
(XLS)

## References

[1] Harris L (2013) What to do about High-Frequency Trading. Financial Analysts Journal 69(2): 6–9.

[2] Dempster MAH, Jones CM (2001) A real-time adaptive trading system using genetic programming. Quantitative Finance 1: 397–413.

[3] Fama E (1970) Efficient capital markets: a review of theory and empirical work. Journal of Finance 25(2): 383–417.

[4] Hasan MZ, Kamil AA, Mustafa A, Baten MA (2012) Stochastic Frontier Model Approach for Measuring Stock Market Efficiency with Different Distributions. PLoS ONE 7(5): e37047. 22629352 10.1371/journal.pone.0037047 PMC3355172

[5] Alvarez-Ramirez J, Rodriguez E, Espinosa-Paredes G (2012) Is the US stock market becoming weakly efficient over time? Evidence from 80-year-long data. Physica A 391: 5643–5647.

[6] Abounoori E, Shahrazi M, Rasekhi S (2012) An investigation of Forex market efficiency based on detrended fluctuation analysis: A case study for Iran. Physica A 391: 3170–3179.

[7] Kim JH, Shamsuddin A, Lim KP (2011) Stock return predictability and the adaptive market hypothesis: Evidence from century-long U.S. data. Journal of Empirical Finance 18: 868–879.

[8] Kim JH, Shamsuddin A (2008) Are Asian stock markets efficient? Evidence from new multiple variance ratio tests. Journal of Empirical Finance 15: 518–532.

[9] Dragota V, Stoian A, Pele DT, Mitrica E, Bensafta M (2009) The development of the Romanian capital market: evidences on information efficiency. Romanian Journal of Economic Forecasting 10(2): 147–160.

[10] Armeanu D, Balu O (2008) Testing the efficiency of Markowitz model on Bucharest Stock Exchange. Economic Computation and Economic Cybernetics Studies and Research 42(1–2) 201–217.

[11] Charles A, Darné O, Kim JH (2012) Exchange-rate return predictability and the adaptive markets hypothesis: Evidence from a major foreign exchange rates. Journal of International Money and Finance 31: 1607–1626.

[12] Charles A, Darné O (2009) The random walk hypothesis for Chinese stock markets: Evidence from variance ratio tests. Economic Systems 33: 117–126.

[13] Trolle AB, Schwartz ES (2010) Variance risk premia in energy commodities. Journal of Derivatives 17: 15–32.

[14] Rosillo R, de la Fuente D, Brugos JAL (2013) Technical analysis and the Spanish stock exchange : testing the RSI, MACD, momentum and stochastic rules using Spanish market companies. Applied Economics 45(12): 1541–1550.

[15] Shynkevich A (2012) Performance of technical analysis in growth and small cap segments of the US equity market. Journal of Banking & Finance 36: 193–208.

[16] Metghalchi M, Marcucci J, Chang YH (2012) Are moving average trading rules profitable? Evidence from the European stock markets. Applied Economics 44(12): 1539–1559.

[17] Grölund A, Yi IG, Kim BJ (2012) Fractal profit landscape of the Stock Market. PLoS ONE 7(4): e33960. 22558079 10.1371/journal.pone.0033960 PMC3338730

[18] Roberts MC (2005) Technical analysis and genetic programming: constructing and testing a commodity portfolio. Journal of Futures Markets 25: 643–660.

[19] Park CH, Irwin SH (2010) A reality check on technical trading rule profits in the US futures markets. Journal of Futures Markets 30: 633–659.

[20] Szakmary AC, Shen Q, Sharma SC (2010) Trend-following trading strategies in commodity futures: a re-examination. Journal of Banking and Finance 34: 409–426.

[21] Allen H, Taylor MP (1992) The use of technical analysis in the foreign exchange market. Journal of International Money and Finance 11: 304–314.

[22] Cheung YW, Chinn MD (2001) Currency traders and exchange rate dynamics: a survey of the US market. Journal of International Money and Finance 20: 439–471.

[23] Olson D (2004) Have trading rule profits in the currency markets declined over time? Journal of Banking and Finance 28: 85–105.

[24] Qi M, Wu Y (2006) Technical trading-rule profitability, data snooping, and reality check: evidence from the foreign exchange market. Journal of Money, Credit and Banking 38: 2135–2158.

[25] Ready MJ (2002) Profits from technical trading rules. Financial Management 31: 43–61.

[26] Hsu PH, Kuan CM (2005) Reexamining the profitability of technical analysis with data snooping checks. Journal of Financial Econometrics 3: 606–628.

[27] Savin G, Weller P, Zvingelis J (2007) The predictive power of “Head-and- Shoulders” price patterns in the US stock market. Journal of Financial Econometrics 5: 243–265.

[28] Kwon KY, Kish RJ (2002) A comparative study of technical trading strategies and return predictability: an extension of Brock, Lakonishok, and LeBaron (1992) using NYSE and NASDAQ indices. Quarterly Review of Economics and Finance 42: 611–631.

[29] Hsu PH, Hsu YC, Kuan CM (2010) Testing the predictive ability of technical analysis using a new stepwise test without data snooping bias. Journal of Empirical Finance 17: 471–484.

[30] De Grauwe P, Decupere D (1992) Psychological barriers in the foreign exchange market. Journal of International and Comparative Economics 1: 87–101.

[31] Osler CL (2000) Support for resistance: technical analysis and intraday exchange rates. Federal Reverse Bank of New York Economic Policy Review 6: 53–68.

[32] Holland JH (1962) Outline for a logical theory of adaptive systems. Journal of the Association for Computing Machinery 3: 297–314.

[33] Allen F, Karjalainen R (1999) Using genetic algorithms to find technical trading rules. Journal of Financial Economics 51: 245–271.

[34] Mendes L, Godinho P, Dias J (2012) A Forex trading system based on a genetic algorithm. Journal of Heuristics 18: 627–656.

[35] Deng S, Sun Y, Sakurai A (2012) Robustness Test of Genetic Algorithm on Generating Rules for Currency Trading. Procedia Computer Science 13: 86–98.

[36] Neely C, Weller P, Dittmar R (1997) Is Technical Analysis in the Foreign Exchange Market Profitable? A Genetic Programming Approach. Journal of Financial and Quantitative Analysis 32: 405–426.

[37] Sullivan R, Timmermann A, White H (1999) Data-snooping, Technical Trading Rule Performance, and the Bootstrap. The Journal of Finance 54: 1647–1691.

[38] White H (2000) A reality check for data snooping. Econometrica 68: 1097–1126.

